# Modulation of innate immunity in airway epithelium for host-directed therapy

**DOI:** 10.3389/fimmu.2023.1197908

**Published:** 2023-05-12

**Authors:** Iwona T. Myszor, Gudmundur Hrafn Gudmundsson

**Affiliations:** ^1^ Faculty of Life and Environmental Sciences, Biomedical Center, University of Iceland, Reykjavik, Iceland; ^2^ Department of Laboratory Medicine, Karolinska Institutet, Stockholm, Sweden

**Keywords:** innate immunity, airway epithelium, microbiota metabolites, epigenetics, innate immune memory

## Abstract

Innate immunity of the mucosal surfaces provides the first-line defense from invading pathogens and pollutants conferring protection from the external environment. Innate immune system of the airway epithelium consists of several components including the mucus layer, mucociliary clearance of beating cilia, production of host defense peptides, epithelial barrier integrity provided by tight and adherens junctions, pathogen recognition receptors, receptors for chemokines and cytokines, production of reactive oxygen species, and autophagy. Therefore, multiple components interplay with each other for efficient protection from pathogens that still can subvert host innate immune defenses. Hence, the modulation of innate immune responses with different inducers to boost host endogenous front-line defenses in the lung epithelium to fend off pathogens and to enhance epithelial innate immune responses in the immunocompromised individuals is of interest for host-directed therapy. Herein, we reviewed possibilities of modulation innate immune responses in the airway epithelium for host-directed therapy presenting an alternative approach to standard antibiotics.

## Introduction

The airway epithelium of the respiratory tract is constantly exposed to particles and microbes inhaled with each breath that could possibly endanger the host. Hence, the highly specialized system of the host innate immune defenses is indispensable, as it keeps pathogens at bay and can limit the damaging effect of environmental pollutants. The airway epithelial cells together with stromal cells and tissue-residing immune cells shape immune responses in the local environment. Those immune responses protect the host from invading pathogens and maintain the local tissue homeostasis by producing signals for cell renewal and regeneration upon damage (1–4). In the first part of this review article, we provided an overview on innate immune functions of the airway epithelial cells covering recent developments like the identification of new cell types by single-cell transcriptomics. In the second part, we described pathogens strategies to subvert host front-line defenses followed by the third part, where we reviewed research on how pathogens subversion mechanisms can be circumvented through modulation of host epithelial innate immune responses by different inducers for host-directed therapy.

## Innate immunity components of the human respiratory epithelium

The airway epithelial cells are central players in the communication between the host and the external environment and together with stromal and immune cells present in local tissues, shape immune responses during homeostasis and disease. This is possible because of a highly complex innate immune system consisting of several components that we review in this section. All those components work together to provide protection of the host mucosal surfaces from the external environment and tissue regeneration upon damage (1–4).

### Human airway and alveolar epithelium

**Figure 1:**
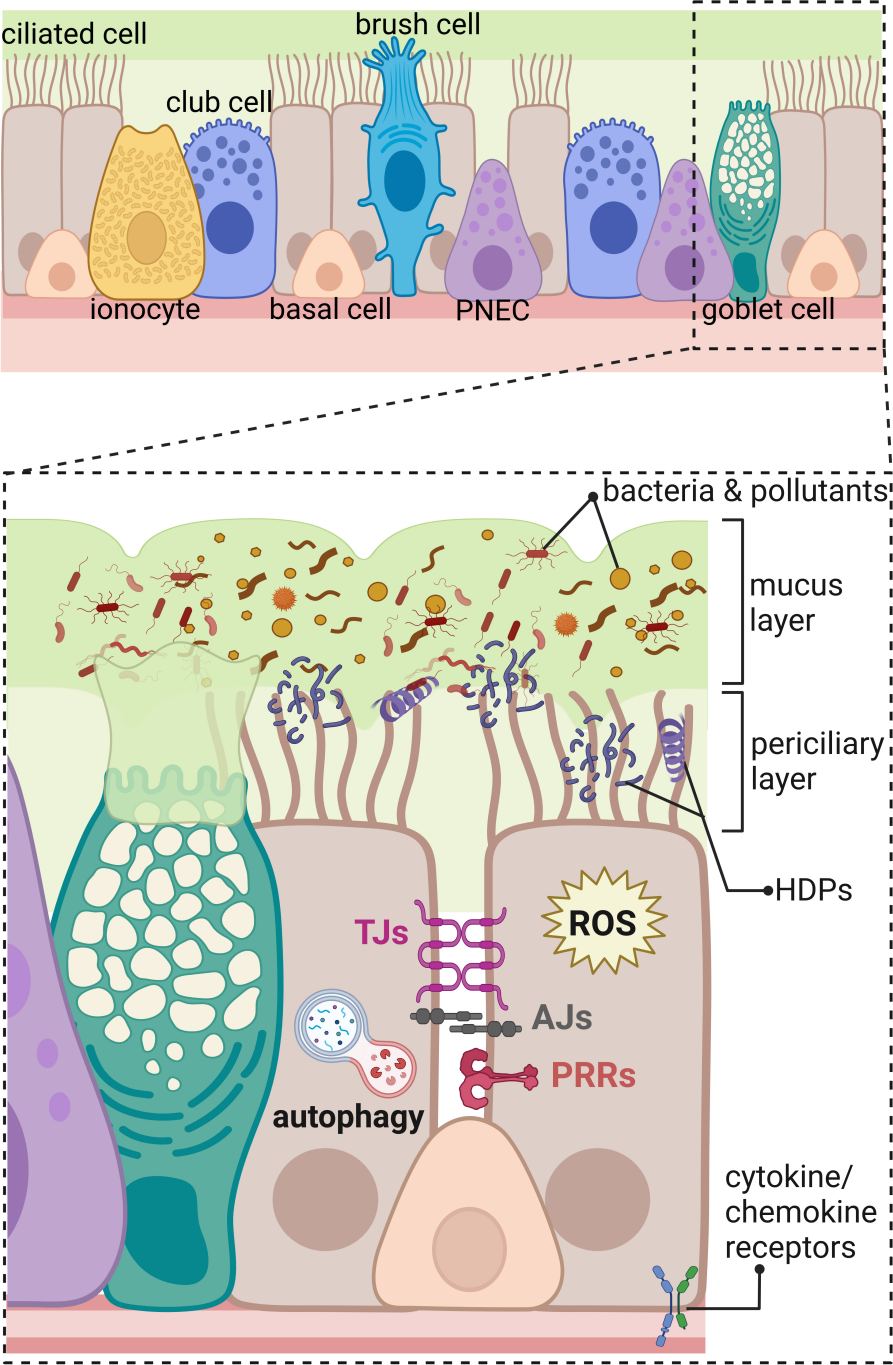
Innate immunity components of the human respiratory epithelium in the distal part of the respiratory tract. The upper panel shows an overview on the different types of cells composing respiratory epithelium of the distal part of the airways including ciliated cells, ionocytes, club cells, basal cells, brush (tuft) cells, pulmonary neuroendocrine cells (PNEC), and goblet cells. The lower panel represents magnified area showing a part of the respiratory epithelium with innate immunity components marked; beginning from the top: mucus layer with host defense peptides (HDPs) entrapping bacteria, and pollutants removed by beating cilia in the periciliary layer. The constant renewal of the mucus layer is provided by goblet cells. Transport of molecules through the paracellular space between neighboring cells is regulated by tight junctions (TJs) and adherens junctions (AJs). Danger signals are recognized by pathogen recognition receptors (PRRs) and cytokine/chemokine receptors. Intracellular components of the innate immunity in the respiratory epithelium include production of reactive oxygen species (ROS) and autophagy eliminating intracellular intruders and particles. Created with BioRender.com.

The airway epithelium lining the upper and lower respiratory tract is composed of different types of epithelial cells forming a single-cell layer of pseudostratified epithelium with tight physical links to the basal lamina and communicating with underlaying stromal cells, such as fibroblasts, airway smooth muscle cells, peri-endothelial (pericytes), and endothelial cells embedded in the connective tissue matrix (2, 4, 5). The four major types of cells in the airway epithelial layer are secretory club cells, goblet cells, ciliated cells, and basal cells (**Figure 1**) (3). Secretory club cells are columnar non-ciliated cells producing factors responsible for the neutralization of inhaled toxic substances and displaying immunomodulatory functions (6). In a mouse model, club cells were shown to act as self-renewing stem cells and as progenitors for ciliated cells that constitute the majority in the airway epithelium (7, 8). Club cells can also differentiate to mucus-producing goblet cells in response to allergens (9). Basal cells are small cuboidal cells that replenish different types of mature cells maintaining epithelial cell turnover during homeostasis and regenerating damaged epithelial barrier (10, 11). In respect to the important role in self-renewal and tissue regeneration, the basal cells seem to be protected from directed exposition to the lumen of the airways by other epithelial cells (**Figure 1**). Additional protection is provided by advanced innate host defense mechanisms. The proportion of each cell type occurring in human airways varies depending on the diameter of the airways, which results in diverse innate immune responses at different levels of the anatomical and histological organization of the respiratory system. For instance, mucus-secreting goblet cells occurring in submucosal glands of the human trachea and large bronchus are replaced by secretory club cells in terminal bronchioles (8, 12). The submucosal glands are composed of four major types of the airway epithelial cells and myoepithelial cells that together form a sophisticated machinery releasing fluids, mucus, and antimicrobial effectors into the luminal space of the airways (1).

Additional types of the airway epithelial cells have been recently described (**Figure 1**) based on the increasing accessibility of the single-cell RNA sequencing, changing our view on the cellular landscape of the airway epithelium that is more heterogeneous than previously thought (3–5, 13). Rare types of airway epithelial cells were distinguished including brush cells (also called tuft cells) and solitary chemosensory cells identified in the human trachea and nasal cavity, respectively (14). The function of brush cells in humans remains largely uncharacterized and, based on the mouse studies, indicates regulation of type II immune responses, such as release of IL-25 in the nasal epithelium and protective reflexes, such as sneezing (14, 15). Interestingly, brush cells do not occur in the healthy human alveolar epithelium. Expansion of brush cells in the upper airways and their *de novo* formation in the lower airways were observed after viral infections with severe acute respiratory syndrome coronavirus 2 (SARS-CoV-2) and influenza A subtype H1N1, respectively (15). Chemosensory cells in the human nasal epithelium detect and mediate reflex reactions in response to irritants and pathogens to prevent their passage down to the lower airways (16). Another rare cell type in the human airway epithelium are pulmonary neuroendocrine cells (PNECs) clustered into neuroendocrine bodies (NEB) (2, 17). Apart from olfactory epithelium in the nasal cavity (18), PNECs are the only cells in the airways supplied with nerves; therefore, they participate in the neurotransmission, act as sensory cells for the oxygen level, and detect and respond to inhaled allergens (2, 17). Another recently identified rare cell types are the ionocytes, showing high expression level of cystic fibrosis transmembrane receptor (CFTR), regulating the ionic microenvironment of the mucus (19, 20). In addition, novel subtypes of progenitor cells were identified, such as precursors of ciliated cells—deuterosomal cells containing numerous centrioles and mucous-ciliated differentiation intermediate cells (3). Moreover, the population of basal cells is more heterogeneous than what was thought. The new intermediate cells were distinguished between basal and 1) club cells—suprabasal cells, 2) PNEC, ionocytes, and brush cells—parabasal cells, and 3) luminal secretory cells—Hillock cells (3, 5, 11).

The distal part of human airways ends with terminal bronchioles mainly composed of club and ciliated cells with very few rare types of cells and almost no basal cells present (8). One can argue that only few basal cells are present in the distal part of human airways because, in principle, the portion of air reaching alveoli should be warmed, moistened, and cleared from pollutants, as the vital function of the alveolar epithelium is the gas exchange provided by epithelial alveolar type 1 cells (also known as AT1, ATI, and type I pneumocytes) (2). Frequently, these defense mechanisms of the proximal part of the respiratory system are not sufficient. Therefore, alveolar innate immune defense mechanisms are activated to protect the host, such as surfactant proteins produced by alveolar type 2 cells (also known as AT2, ATII, and type II pneumocytes), that also function as progenitor cells for AT1 cells in the adult lung (1, 2, 21). Overall, the recent advances in transcriptomic and proteomic analyses opened a new avenue for further characterization of innate immune functions of new epithelial cells subtypes and the signaling pathways guarding their differentiation.

### Host defense peptides

Numerous antimicrobial effectors are produced by the airway epithelial cells for effective host innate defenses. Among them are host defense peptides (HDPs) (**Figure 1**), previously commonly called antimicrobial peptides (AMPs), divided into two main families of cathelicidins and defensins (22–24). HDPs can be produced constitutively and induced upon stimuli. Their constitutive expression can be enhanced upon stress conditions, like infection or injury. Most of HDPs are stored in the cellular granules as a pro-form, which is further released and processed upon a danger signal (22, 25). Human cathelicidins have one dominant mature peptide LL-37 encoded by *CAMP* gene (cathelicidin antimicrobial peptide). Human cathelicidin is produced as a pro-form (hCAP18; pro-LL-37) containing N-terminal cathelin domain that is cleaved off by serine proteases, such as kallikreins in the skin and proteinase 3 from neutrophiles to release the mature LL-37 peptide (25–28). LL-37 has an α-helical structure and a cationic, amphipathic character and can be processed in the skin to shorter peptides (26, 29). Human cathelicidin is constitutively expressed at rather low protein levels in airway epithelial cells showing only the pro-form to be secreted when the cells are cultured *in vitro*. The expression of cathelicidin is enhanced upon stimuli, indicating a highly responsive inducible system for host defense (30–32). During inflammation, the migration of neutrophiles to the site of infection contributes to the processing of the pro-form hCAP18 to the active LL-37 peptide in the lung, as neutrophiles are loaded with proteinase 3 and other enzymes. However, whether the processing of constitutively expressed pro-form hCAP18 to the active LL-37 peptide takes place on the airway epithelial surfaces in physiological conditions during homeostasis remains to be further elucidated, as the *in vitro* studies with the airway epithelial cells do not closely recapitulate the *in vivo* environment. On the other hand, the analysis of the airway surface liquid (ASL) indicated the presence of mature LL-37 (33). However, one may argue about fact that the source of mature LL-37 peptide in ASL might come from immune cells present in lungs. Therefore, the dynamics of the cathelicidin processing to the mature LL-37 peptide in the homeostatic conditions of the lung epithelium remains to be further investigated, similar to the identification of potential lung tissue specific enzymes responsible for the cleavage and confirmation if LL-37 is processed to shorter peptides with characterization of their potential role in shaping local lung innate immune responses. In contrast to cathelicidins, human defensins have several members and are divided in α-, β-, and θ-defensins (34). Alpha- and beta-defensins are produced in the airway epithelium constitutively and inducible as inactive precursors that undergo further processing to the active form (21, 22, 35, 36). In human genome, there are genes encoding the third group of θ-defensins; however, proteins are not produced because of premature codon stop (37). The expression of antimicrobial effectors in each cell type present in the airway epithelium has been explored and is available in Human Protein Atlas (HPA). According to HPA, the main sources of cathelicidin in bronchus are basal respiratory cells (https://www.proteinatlas.org/ENSG00000164047-CAMP/single+cell+type/bronchus).

The primary function of the HDPs is their bactericidal effect at the range of micromolar concentrations on both Gram-positive and Gram-negative bacteria, but they are also effective against viruses and fungi (26, 35, 38). The common antimicrobial activity of HDPs is associated with their cationic and amphipathic properties. Positively charged HDPs can strongly interact through electrostatic and hydrophobic interactions with negatively charged phospholipids of the bacterial cell membrane and pathogen-associated molecular patterns (PAMPs), such as LPS of Gram-negative bacteria (39, 40). The interaction of HDPs with the bacterial membrane causes disruption, leakage of the intracellular components, and bacterial cell death (24). Another, less known antimicrobial mechanism of HDPs is the translocation of peptides through the membrane and binding to bacterial intracellular targets, for example, cardiolipin resulting in bacterial cell death (41). Furthermore, human β-defensin 3 has been shown to disrupt bacterial cell wall biosynthesis by binding to lipid II, which makes bacteria more vulnerable to damage (42). Hence, peptides have different activities against bacteria, but the membrane disruption seems of a major general importance. In case of the viral infections, HDPs bind and destabilize viral structures like viral envelope of influenza viruses, respiratory syncytial virus (RSV), Zika virus (38, 43, 44), and viral capsid of rhinoviruses (45). Similar mechanism takes place during fungal infections, like with *Candida albicans*, where HDPs permeabilize yeast cell membrane (46). Of note, at physiological concentrations, the HDPs do not damage human cell membranes due to its lack of negative charge in the outer leaflet of the membrane and presence of cholesterol (47). However, HDPs at higher concentrations might be able to damage host’s cells, for instance when granulocytes are recruited to the site of infection in the lung (48, 49). Interestingly, recently, it has been described that the fragments of LL-37 peptide (17–29 aa residues) can cluster together to form highly organized oligomers resembling fibril-like tubules of unknown function (50). Some pathogens, such as *Staphylococcus aureus*, produce similar tubules what is recently discussed as an example of the bacterial mimicry, perhaps to evade host antimicrobial responses potentially exerted by such HDPs fibril-like oligomers (51). However, the existence and physiological relevance of such LL-37 fibrils in the lung epithelial surfaces have not been described so far.

Apart from antimicrobial activity, HDPs display immunomodulatory functions (24). HDPs are chemoattractant for immune cells, e.g., human β-defensin 2 (hBD2) is a chemoattractant for mast cells (52) and LL-37 for neutrophils, monocytes, and T cells (53, 54). HDPs also indirectly recruits leukocytes to the local site of infection or injury by inducing release of chemokines and cytokines (24). For instance, defensins can induce expression of proinflammatory IL-8, a chemoattractant for neutrophils, in human A549 lung epithelial cell line and therefore display proinflammatory function *in vitro* (55). HDPs interfere with the cell signaling cascades while displaying at the same time a dual pro- and anti-inflammatory role, depending on the local environment and the phase of infection. During infection when nuclear factor kappa B (NF-κB) pathway is activated by bacterial LPS binding Toll-like receptor 4 (TLR4) receptor, LL-37 peptide can selectively inhibit production of proinflammatory tumor necrosis factor (TNF) and reactive oxygen species (ROS) while at the same time stimulate IL-8 production in epithelial cells to attract immune cells (56). HDPs have pro- and anti-inflammatory effects that seem to be exerted depending on the stage of infection. In the early stage of bacterial infection with *Pseudomonas aeruginosa*, LL-37 enhances proinflammatory response in airway epithelial cells (57). Cathelicidins are also able to prevent activation of TLR2 and TLR4 signaling in macrophages by non-viable bacteria and their products at later stage of infection, resulting in reduced production of proinflammatory response that might protect local tissue from the injury (58). Therefore, HDPs can play a dual role in shaping both pro- and anti-inflammatory response and maintaining tissue homeostasis. HDPs also display additional functions by enhancing phagocytosis, ROS production, and participation in neutrophil extracellular trap (NET) formation, contributing to enhancement of the bacterial clearance (24). HDPs also initiate T-cell response by promoting Th17 differentiation (59) and could, therefore, play a role in the intersection of innate and adaptive immunity. Furthermore, local environment also affects immunomodulatory function of HDPs by their post-translational modifications (PTMs). The citrullinated LL-37 peptide was detected in human bronchoalveolar lavage; however, citrullination affected the net charge of the peptide that lost the ability for bacterial killing (60). Another evidence is citrullination of LL-37 by peptidyl arginine deaminase, where the peptide loses its ability to enhance proinflammatory response in macrophages (61). These studies indicate that PTMs regulate the mode of action of HDPs for the maintenance of the local tissue homeostasis, and future exploration of how additional PTMs affect function of HDPs in lungs is of interest.

Apart from HDPs, there are also several antimicrobial proteins like lysozyme, degrading bacterial peptidoglycans, and bacteriostatic lipocalin 2 and lactotransferrin (21). The bacteriostatic effect of lipocalin 2 and lactoferrin are linked to the inhibition of iron uptake by bacteria from the local environment, as they bind bacterial iron-chelating molecules. Lipocalin 2 has been shown to be effective against *Escherichia coli* causing pneumonia (62), and mutant mice lacking lipocalin 2 were more susceptible to *Klebsiella pneumoniae* infections (63). Similarly, S100A7 (psoriasin) protein from S100 protein family containing several antimicrobial effectors (e.g., S100A8/9 protein known as calprotectin) has been shown to kill *E. coli* by Zn^2+^ chelation (64). Airway epithelial cells and alveolar macrophages constitutively express S100A7 that is enhanced upon *S. aureus* challenge (65). Interestingly, the mechanical strain generated during breathing enhances expression of S100A7 protein in alveolus-on-chip model through activation of mechanosensitive ion channel TRPV4 (transient receptor potential vanilloid-type 4). Moreover, when TRPV4 and the S100A7 target receptor—receptor for advanced glycation end products (RAGE)—were blocked by inhibitors, the viral load of H3N2 influenza was increased, demonstrating the importance of S100A7 for lung defenses (66). Furthermore, antiproteases like secretory leukocyte protease inhibitor (SLPI) and elafin exhibit antimicrobial properties against pathogens *P. aeruginosa* and *S. aureus* in lung epithelial cells (67–69) and have anti-inflammatory potential, e.g., by inhibition of NF-κB pathway through reducing degradation of IκBα in macrophages and endothelial cells (70). Ribonuclease 7 (RNase7) discovered in the skin where it displays antimicrobial effects against several pathogens has also been shown to be expressed in human respiratory tract (71). Interestingly, the primary source of RNase7 in human airways are basal cells that express RNase7 upon stimulation with inactivated *H. influenzae* and cigarette smoke, therefore indicated as a second line of front-line defenses in case of injury of mature differentiated epithelial cells (72). Similarly, RNase7 has been induced in airway epithelial cells upon infection with Mtb, where it marked intracellular bacteria to a limited extent. However, the direct effect of RNase7 on elimination of Mtb was not shown and requires further investigation (73). Additional components of the innate immunity in the lung epithelium are collectins, surfactant protein A and D (SP-A and SP-D), produced by alveolar type 2 cells (21). They tag bacteria for opsonization to increase phagocytosis by the alveolar macrophages (74). The immunomodulatory function of collectins can be exemplified by the inhibitory effect of the SP-A on the production of IL-8 by eosinophils present during allergic response (75). The antimicrobial and immunomodulatory functions are also displayed by plate-lung-nasal-clone (PLUNC) proteins shown to have antibacterial effect against *Mycoplasma pneumoniae* and reduce production of proinflammatory cytokine IL-8 (76). Of note, several chemokines like CXCL9 and CXCL11, present in the lung epithelium as a result of interferon gamma (IFNγ) stimulation, have antibacterial functions against *E. coli* and *L. monocytogenes* (77). Host defense effectors produced by club cells such as club cell protein 10 (CC-10) inhibit NF-κB signaling pathway and production of proinflammatory cytokines and chemokines in bronchial epithelial cells (78). Similar to CC-10, club cell secretory protein (CCSP) was shown to reduce inflammation and viral load during RSV infection (79). Next, the levels of secretoglobin A1A (SCGB1A1) also produced by club cells were reduced in the airways of patients with asthma in comparison to healthy individuals (80). Allergen-specific immunotherapy increased expression of SCGB1A1 considered as anti-inflammatory mediator in the lower airways (81). Secretoglobin A1A was also shown to affect alveolar macrophages by attenuation of the surge of inflammatory cytokines during activation of TLR receptors. Deficiency of secretoglobin A1A facilitated development of proinflammatory M1 phenotype of pulmonary macrophages, indicating the importance of physiological levels of SCGBA1A for innate immune defense and maintenance of local tissue homeostasis (82). Another important indirect link to HDP activity is the expression and function of CFTR and the non-gastric H^+^/K^+^ adenosine triphosphatase (ATP12A) in lung epithelial cells, including recently identified ionocytes. Both CFTR and ATP12A regulate pH of the airway surface liquid (ASL) by secretion of ${\text{HCO}}_{3}^{-}$ and H^+^, respectively, that is detrimental for antimicrobial activity of HDPs (83, 84).

A further important element of the innate immunity in the airway epithelial cells is the production of reactive oxygen species (ROS) (**Figure 1**). For many years, ROS were attributed to the lung tissue damage and tissue aging as a result of the oxidative stress (85, 86) but in fact ROS play an important function in the elimination of invading pathogens (21). Dual oxidases (DUOX) are key enzymes responsible for the generation of ROS in the lung epithelium, including superoxide and hydrogen peroxide, for effective pathogen elimination (87, 88). In addition, the protective role of NOX1 activity have been shown to limit inflammatory response and lung tissue damage exerted by influenza A at early stage of infection in mice (89) even though the NOX1 activity in different circumstances, such as hyperoxia, can cause tissue damage (85). The view on ROS generation and redox signaling in epithelial cells is developing to better understand their role in pathological situations and demonstrate that the physiological levels of ROS are important for cellular signaling during homeostasis (86, 90).

### Mucus and mucociliary clearance

A physical component of the innate immune system is the airway surface liquid (ASL) composed of the mucus and periciliary layer on the airway epithelium (**Figure 1**) (91). The ASL provides a physical and chemical barrier for the invading pathogens and inhaled particles protecting the airway tissue from the damage (92). The chemical barrier of ASL is provided by the low salt content (93), pH maintained by ${\text{HCO}}_{3}^{-}$ and H^+^ (94, 95), and hydration maintained by Na^+^ and Cl^−^ ion gradient (96). This environment facilitates the formation of MUC5B bundles (12) and keeps HDPs active for killing of entrapped pathogens in the mucus layer (83, 84) for their removal by mucociliary clearance of beating cilia (21, 93). The periciliary layer of ASL composed of transmembrane mucins and periciliary liquid reduces the friction of the constantly moving mucus that clears airways from bacteria and pollutants during homeostasis (12). The dynamic process of mucociliary clearance keeps the airways almost devoid of bacteria in healthy individuals (12, 93) causing the characterization of any existing lung microbiome very difficult and so far confirmed only by detection of bacterial nucleic acids (97, 98). In addition, the anaerobic bacterial fermentation products were detected in the lungs of patients with HIV (99, 100); however, those ground-breaking evidence shall be carefully interpreted and further confirmed by the characterization of additional microbiota metabolites and their original source of production. Impairment of the mucociliary clearance by primary ciliary dyskinesia (101) or defects in the function of ion channels in cystic fibrosis (CF) (92) cause clogging of the airways with mucus, creating a favorable environment for bacterial growth (12). Therefore, pre-existing bacteria during chronic respiratory diseases are sometimes referred in the literature as “lung microbiota” (102), although it should be emphasized that this terminology is used in the context of pathological conditions. In general, the studies on the mucus composition and physiology together with the interaction of innate immunity components with mucus are of interest and one of the future directions in the field investigating host defenses.

### Pathogen recognition receptors

One of the initial functions of innate immunity in the airway epithelium is pathogen detection by pathogen recognition receptors (PRRs) (**Figure 1**), recognizing PAMPs. Upon stimulation, PRRs activate signaling cascade leading to the inflammation and clearance of the pathogens. Among different types of the PRRs, present in the airway epithelium are TLRs, NOD-like receptors (NLRs), RIG-I-like receptors (RLRs), C-type lectin receptors (CLRs), and formyl-peptide receptors (FPRs) (21, 103–105).

In the airway epithelium, all types of the TLRs (TLR2/1, TLR2/6, TLR3, TLR4, TLR5, TLR7, TLR8, TLR9, and TLR11) are present, anchored in the cell membrane and endosomes (21). However, the mapping of TLRs with their exact spatial distribution within airway epithelial cell membranes at different regions of the respiratory tract, similar to the study done for the intestinal epithelium (106), is still missing. TLRs recognize bacterial lipopeptides, lipopolysaccharide (LPS), flagellin, DNA, and RNA by the leucine-rich repeat (LRR) motif that is linked through a single transmembrane domain to Toll/IL-1 receptor (TIR) intracellular motif. The activation of the receptor requires binding of the adaptor protein MyD88 or TIR-domain-containing adapter-inducing interferon-β (TRIF) to the TIR domain. MyD88 is an adaptor protein for all TLRs, except TLR3 and TRIF for TLR3 and TLR4. Other adaptor proteins like TIR domain containing adaptor protein (TIRAP), translocation associated membrane protein 1 (TRAM), sterile alpha and TIR motif containing (SARM) have been shown to be involved in the TLR signaling. The stimulation of TLRs initiates NF-κB and mitogen-activated protein kinase (MAPK) downstream signaling, leading to induction of proinflammatory cytokines and type I interferons (21, 107).

Unlike TLRs, apart from cell and endosomal membranes, NLRs occur as soluble receptors in the cytoplasm. In the airway epithelium, NLRs with caspase recruitment domain (CARD) are represented by NOD1 and NOD2 receptors, recognizing bacterial peptidoglycans and working in synergy with TLRs towards activation of NF-κB and MAPK pathways (108). The other types of NLRs, containing PYD (pyrin) and NLRB (baculoinhibitor of apoptosis protein) domains form inflammasome controlling cleavage of IL-1β and IL-18 pro-forms by caspase-1 (21, 103). For instance, in human lung epithelial cells NRLP3 receptor harboring pyrin domain forms NRLP3 inflammasome responsible for cleavage of IL-1β upon *C. albicans* or influenza A virus infection (109, 110).

Human airway epithelial cells have also cytoplasmic receptors for the detection of pathogen’s nucleic acids (21). Among them are RIG-I-like receptors (RLRs), recognizing viral ss/dsRNA of influenza and paramyxoviruses causing respiratory diseases, e.g., RSV (111, 112). The group of RLR receptors include retinoic acid inducible gene-I (RIG-I), melanoma differentiation-associated gene 5 (MDA5) and RIG-I-like receptor dsRNA helicase (LGP2) (103). With the involvement of the adaptor proteins, e.g., mitochondrial antiviral-signaling protein (MAVS), RIG-I and MDA5 receptors transduce signals activating NF-κB, interferon regulatory factor 3 and 7 (IRF3 and IRF7) pathways leading to the production of proinflammatory cytokines and interferons (108). Other cytoplasmic receptors recognize bacterial DNA and bacterial signaling molecules such as cyclic dinucleotides (CDNs) (21). A prominent member of this family is the stimulator of IFN genes (STING) located on the ER membrane. STING recognizes CDNs and is an adaptor protein for other cytoplasmic receptors like interferon gamma inducible protein 16 (IFI16) recognizing *Streptococcus pneumoniae* dsDNA (113). Moreover, STING can by activated by cyclic GMP-AMP (cGAMP) synthetized by cGAMP synthase (cGAS) in human airway epithelial cells upon detection of not only microbial/viral DNA in the cytoplasm but also by self-DNA coming from the nucleus or damaged mitochondria (114).

Additional PRRs are C-type lectin receptors (CLRs) on the airway epithelial cells recognizing carbohydrates present on the pathogens and activating proinflammatory response (21, 103). The CLRs occur as membrane-anchored receptors like Dectin-1 recognizing *Haemophilus influenzae* and *Aspergillus fumigatus* infections (115, 116). The second form of CLRs are soluble collectins containing C-type lectin domain like SP-A and SP-D exhibiting antimicrobial activity through opsonization of bacterial, viral, and fungal pathogens (21). Formyl-peptide receptors (FPRs) are also expressed on the epithelial cells of the respiratory tract, where they recognize not only bacterial formylated peptides but also host-derived stimulants, such as mitochondrial proteins from damaged cells that chemoattract leukocytes promoting inflammation. Host LL-37 has also been shown to activate FPR2 (aka FPRL1) and is considered as a proinflammatory stimulus in chronic obstructive pulmonary disease (COPD). Depending on the local tissue environment, activation of FPRs can have a dual pro- or anti-inflammatory role, and it has been associated with the tissue regeneration and wound healing (105, 117).

Interestingly, it has recently been shown that PTMs of PRRs can be considered as contributing to a fine-tuning mechanism limiting inflammation; for instance, palmitoylation of NLR family pyrin domain containing 3 (NLRP3) prevents activation of inflammasome (118). Studies on how different PTMs affect PRRs downstream signaling in response to pathogens and during inflammation is one of the future directions for research in the host–pathogen interactions field.

### Cell junctional complexes

The structural elements of the airway epithelial barrier integrity are tight (TJs) and adherens junctions (AJs) that determine the polarization of the epithelial cells into the apical and basolateral site providing physical barrier regulating paracellular flux through epithelial layers (119). Hence, the epithelial barrier integrity provided by TJs and AJs can be considered as part of the epithelial defense system (**Figure 1**). TJs and AJs are composed of transmembrane proteins, such as occludin, tricellulin, claudins, and junctional adhesion molecules (JAMs) and many others present in TJs together with E-cadherin and nectins in AJs. Transmembrane proteins of TJs interact with the proteins of the intracellular junctional plaque containing multiple interaction domains, such as zonula occludens-1 (ZO-1)—a well-defined protein from the junctional plaque complex—and its deletion is lethal in the mouse embryos. The examples of AJs intracellular junctional plaque proteins are α- and β- and p120 catenins and afadin (AF6) displaying similar functions. The intracellular junctional plaque proteins are coupled to cytoskeleton proteins, for example to actin through C-terminal domain of ZO-1 (119). The importance of the functional epithelial barrier sealing the deeper tissues from the external environment for the maintenance of the local tissue homeostasis seems to be explained by the epithelial barrier hypothesis (120). It highlights that the leaky epithelial layer caused by disruption of TJs and AJs by detergents, pollutants, allergens, and pathogens (121–125) contributes to increased incidence of allergies, asthma, and autoimmune diseases (120). The exposure to such environmental insults creates a positive feedback loop of further epithelial layer destruction. This process includes a subsequent translocation of the microbiota and opportunistic pathogens through a disrupted epithelial layer to the lamina propria that activates macrophages and T cells, ultimately leading to inflammation (120). During inflammatory response, cytokines cause further disintegration of tight junctions in the airway epithelium (126, 127), exacerbating the inflammatory response that may become a chronic state. Therefore, the disruption of the net of TJs and AJs can be considered as one of the early onsets of the disease. Hence, the maintenance of the epithelial barrier integrity is crucial for balanced innate and adaptive immune responses in the airway epithelium.

### Autophagy

Autophagy is a complex process involving interaction of several different proteins, leading to engulfment of the cytoplasmic cargo during autophagosome formation and its subsequent degradation upon fusion with lysosomes. Autophagy is one of the key cellular processes maintaining balance in cells exposed to a constantly changing environment. It plays a major “housekeeping” function on the cellular level where it is responsible for the degradation of damaged organelles and unfolded/misfolded proteins (128). An important function of the autophagy process in respect to innate immunity is the degradation of invading pathogens, when the initial innate immunity barrier—mucus, HDPs, and TJs—was not sufficient to halt the pathogen entering the cells (129). Pathogenic *P. aeruginosa* (130), conidia of *Aspergillus fumigatus* (131), and influenza A virus (132) can be effectively eliminated or restricted by autophagy in airway epithelial cells. Therefore, autophagy can be considered as part of cell autonomous innate immunity and an intracellular defense mechanism of the “last chance” for the prevention of pathogen dissemination and progression of infectious disease (133). The commonly used cell model to study the role of autophagy in innate immunity are phagocytic professional cells, such as macrophages. However, the importance of autophagy in epithelial cells should be highlighted due to the vital role of epithelial cells in the first line of the host defense and epithelial homeostasis (**Figure 1**). During homeostasis, autophagy can control ciliogenesis and ciliary function by regulating the length of motile cilia in airway cells (134). The biogenesis of primary cilia is regulated by autophagic degradation of centriole and centriolar satellite protein OFD1 (135) and the function of motile cilia by degradation of kinesin family member 19 (Kif19)—an essential protein controlling ciliary length that should be inhibited to maintain the correct length of cilia (136).

Moreover, the importance of autophagy in human lung is demonstrated in CF patients whose autophagy is impaired due to aggregating Beclin-1. In the cell and animal models of CF, this phenotype can be rescued upon Beclin-1 restoration, suggesting a key role of autophagy in the lung homeostasis (137). Autophagy was shown as a central contributor to IL-13-mediated mucus hypersecretion by airway epithelial cells in COPD and asthma (138). Moreover, autophagy regulates apical localization of DUOX1 in airway epithelial cells and ROS production in response to chronic IL-13 exposure (139). Furthermore, autophagy maintains the airway progenitor cells pool and regulates cell differentiation for epithelial regeneration (140). In addition, one of the clinical symptoms of the Hermansky–Pudlak syndrome type 1, a rare genetic disorder impairing vesicle trafficking, is lung fibrosis and impaired innate immune antimicrobial responses due to amplified mechanistic target of rapamycin kinase (mTOR) signaling resulting in reduced bacterial clearance, indicating autophagy as a key cellular process in physiological and pathological conditions (141, 142). Autophagy in the airway epithelium can be considered as a double-edge sword because in particulate-matter-induced airway inflammation, autophagy contributes to the epithelial injury (143). The basic research on the autophagy process and its regulation in different conditions remain future perspectives for the development of novel strategies for treating respiratory tract diseases.

### Epigenetics and innate immunity

**Figure 2:**
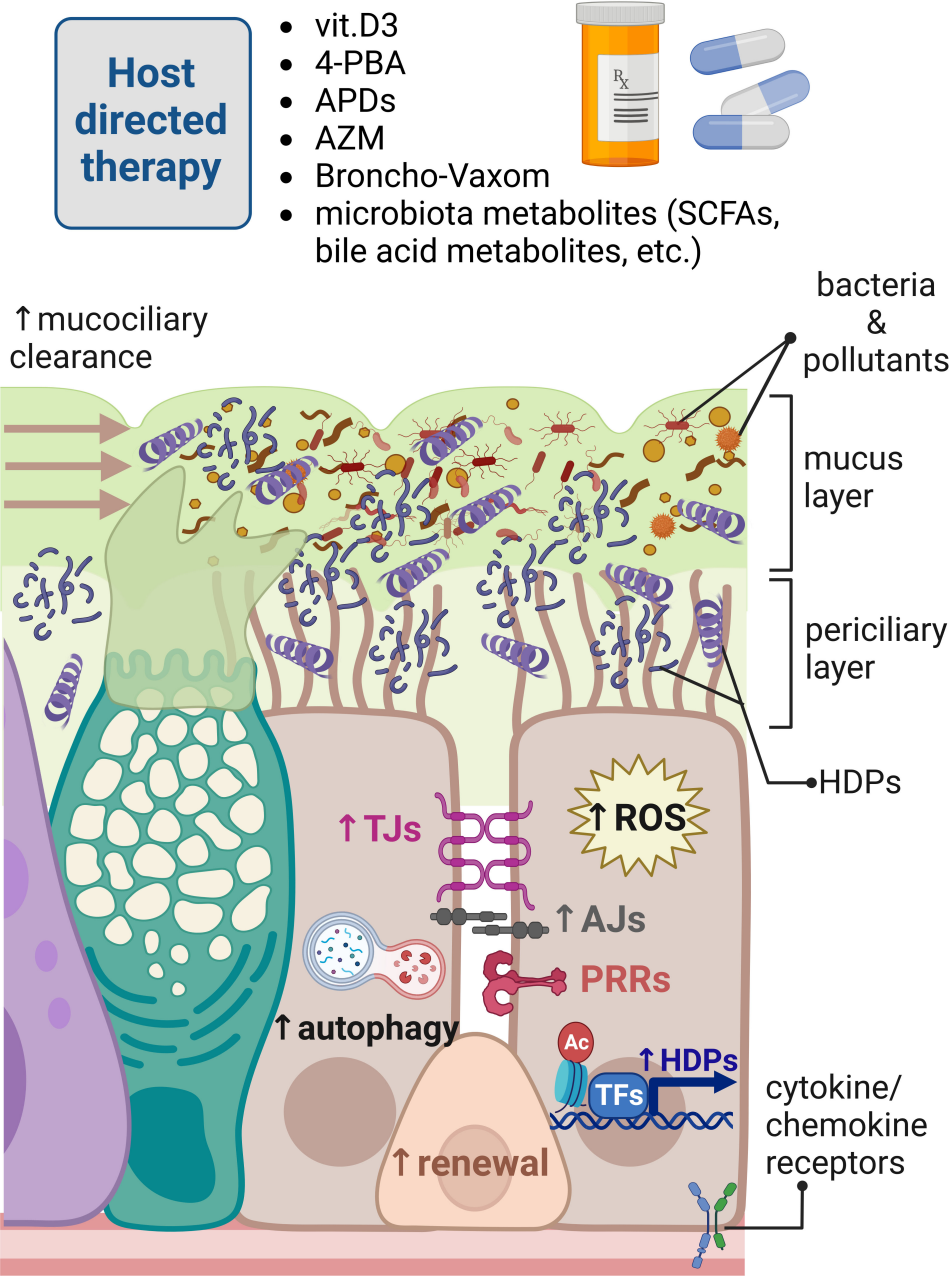
Modulation of innate immune responses in human respiratory epithelium for host-directed therapy. Different innate immunity inducers vitamin D3, 4-phenylbutyrate (4-PBA), aroylated phenylenediamines (APDs), Broncho-Vaxom (bacterial lysates), and microbiota metabolites can boost innate immune responses in the respiratory epithelium fending off pathogens invading mucosal surfaces of the respiratory tract. These responses are exerted by enhancement of the mucociliary clearance, removing dead bacteria and inhaled pollutants neutralized by increased expression of host defense peptides (HDPs) through the epigenetic modulation of histone epigenetic marks and transcriptional regulation. Innate immunity inducers and azithromycin (AZM) can also strengthen epithelial barrier integrity providing physical barrier for invading pathogens. Innate immunity modulators can increase autophagy and production of reactive oxygen species (ROS) for effective elimination of foreign molecules and microbes. Bacterial lysates, such as Broncho-Vaxom, can stimulate pathogen recognition receptors (PRRs), perhaps to train epithelial innate immunity through imprinting innate immune memory in renewing epithelial cells. Created with BioRender.com.

Innate immune responses in the airway epithelium are also regulated by epigenetic modifiers of DNA methylation and post-translational modifications of histones. The epigenetic regulation of gene expression by modulation leading to chromatin opening allows for a rapid response to environmental changes. These processes are tightly regulated by the equilibrium of epigenetic enzymes and their counterparts, for example by histone acetyltransferases (HATs)—histone deacetyltransferases (HDACs) and DNA methyltransferases (DMTs). The importance of the DNA methylation status seems to be highlighted by the studies showing that DNA methylation pattern is changed in respiratory tract diseases, such as increased DNA methylation in *NLRP3* gene of COPD patients (144). On the other hand, decreased methylation in the promoter region of *TLR2* contributed to enhanced inflammatory responses in the airway epithelium of CF patients in response to bacterial peptidoglycan (145). Different PTMs of histones can modulate the front-line innate immune defenses in response to pathogen invasion (**Figure 2**). For example, acetylation of histones allows for more permissive chromatin structure facilitating gene expression. Upon stimulation with LPS, the activation of MAPK pathway leads to phosphorylation of H3S10 and additional acetylation of H3S10K14 at the promoter of *IL12*, leading to chromatin opening for NF-κB and induction of IL-12 expression (146). Furthermore, an increased acetylation of H3K18 was observed in *STAT6* locus in the airway epithelial cells of asthmatic patients (147). Although that study did not show increased STAT6 expression, it is known that STAT6 signaling is affected in asthma and, together with elevated levels of IL-4 and IL-13 driven by Th2 immune responses, leads to mucus hypersecretion in mice, which is a characteristic for asthma, COPD, and CF (148). Moreover, the treatment of asthmatic airway epithelial cells with IL-4 and IL-13 caused the impairment of the airway epithelial barrier integrity concomitant with enhanced expression of HDACs (1 and 9) and sirtuins (SIRT6 and 7). Interestingly, inhibition of HDACs in asthmatic airway epithelial cells by quisinostat (class I and II HDAC inhibitor) improved the epithelial barrier integrity by increasing the expression of TJ proteins (149). On the other hand, inhibition of HDACs for example, by deletion of *Hdac1* in mice exposed to the allergen caused more stable Th2 immune responses resulting in mucus hypersecretion by goblet cells, demonstrating an important role of HDAC1 in allergic diseases (150). The enzyme HDAC2 can suppress NF-κB and AP-1 signaling leading to the inhibition of proinflammatory response caused by stimulation of TLR4. Interestingly, the downregulation of HDAC2 in lungs of COPD patients and rats exposed to cigarette smoke suggest that HDAC2 expression is modulated in response to environmental factors and protects from inflammation and mucus hypersecretion during homeostasis (148, 151, 152). Unlike HDAC2, exposition to cigarette smoke increased expression of HDAC6 enhancing autophagy. Rapid protein turnover due to increased autophagy led to shortening of the ciliary length that contributes to impaired mucociliary clearance observed in COPD. These effects were diminished in *Hdac6^−/Y^* mice exposed to cigarette smoke, suggesting the inhibition of HDAC6 as a potential therapeutic target for COPD treatment (153, 154). Apart from HDAC6, many epigenetic modifiers are suggested to regulate autophagy processes (155), such as EZH2 methyltransferase responsible for the trimethylation of H3K27 shown to inhibit autophagy (156). The importance of EZH2 was demonstrated to regulate differentiation of airway epithelial stem cells that is aberrant in *Ezh2*-deficient mice resembling an altered airway epithelial cell differentiation typical for COPD (157). Overall, the epigenetic regulation of innate immune responses is intensively investigated, and the studies showing epigenetic regulation of HDPs in the lung epithelium are ongoing (158).

## How pathogens subvert host innate immune defenses in lungs

Efficient innate immune responses in airway mucosa are essential for maintaining respiratory functions. The mucosal surface of the airways is also an initial site for pathogens interaction with the host. Many pathogens can subvert airway mucosal defense mechanisms and cause disease, especially in immunocompromised individuals. In this section, we will discuss those strategies based on the selected examples of respiratory pathogens that mainly concentrate on bacteria, as those were extensively studied throughout the years in comparison to viruses and fungi. Viral subversions mechanisms of host innate immune responses in lungs has recently gained more attention due to SARS-CoV-2 pandemic, while little is known about fungal strategies that seems to be relevant, especially in the development of severe fungal infections in immunocompromised individuals.

### Bacteria

*Pseudomonas aeruginosa* (Pa) is an opportunistic pathogen usually harmless for healthy people; however, it can cause pneumonia in vulnerable immunocompromised individuals like patients with chronic respiratory diseases, such as CF, COPD, and hospitalized patients with supportive mechanical ventilation. Pa is known to use different strategies and virulence factors for the effective colonization of the host mucosal surfaces (159). The bacteria utilize flagellin for movement and pili for adhesion to the host cells. Once the host–pathogen adhesion is established, Pa injects bacterial toxins, such as ExoS to the host cells by type 3 secretion system (T3SS) causing acute infection (160). Furthermore, lung tissue damage in pneumonia is caused by disruption of the airway TJs and AJs integrity by Pa elastase that also degrades collectins (SP-A and SP-D), collagen, complement components, and LL-37 (159, 161). Pa rhamnolipids also disrupt TJs integrity in the airway epithelium facilitating Pa invasion (162). As an additional virulence factor, pyocyanin impairs ciliary function and inhibits host catalase, which contributes to the oxidative lung damage (163, 164). Furthermore, Pa competes for the iron source with host antimicrobial effectors, necessary for the activity of antimicrobial effector, lactoferrin, in the airway epithelium (165). Depending on the local environment of the host airway epithelium, especially during mucus clogging in CF and COPD, Pa can switch strategy of host colonization, from the invasive to a more adaptative one by formation of the biofilm. Quorum-sensing mechanisms allow for the communication of bacteria thriving in the biofilm and involve bacterial molecules, such as acyl homoserine lactones (AHL). At the same time, bacterial molecules involved in quorum sensing affect host cells; for example, AHL facilitates apoptosis of neutrophiles but not the host’s airway epithelial cells (166). In addition, 2-aminoacetophenone enhances the host’s HDAC1 activity suppressing proinflammatory response that facilitates bacterial survival (167). Moreover, Pa in biofilm produces alginate, a mucopolysaccharide used for bacterial encapsulation increasing bacterial fitness to persist on the mucosal surfaces of CF patients (168). These bacterial biofilm subversion and evasion mechanisms of the host’s innate immune defenses facilitate chronic Pa infection that can be very difficult to eliminate by antibiotics and contributes to development of antibiotic resistance. Many opportunistic pathogens have gained resistance to antibiotics routinely used for the treatment of infections. Therefore, Pa has been included in a group of ESKAPE pathogens (ESKAPE stands for the group of pathogens: *Enterococcus faecium*, *Staphylococcus aureus*, *Klebsiella pneumoniae*, *Acinetobacter baumanii*, *Pseudomonas aeruginosa*, and *Enterobacter* spp.) that cause nosocomial infections (169).

*Klebsiella pneumoniae* (Kp) is another pathogen from the ESKAPE group causing pneumonia. Kp is well-known to contain several antibiotic resistance genes, including extended-spectrum beta-lactamase (ESBL), and carbapenemase encoding genes, which is referred to as a multidrug-resistant (MDR) Kp. Apart from gaining antibiotic resistance genes, Kp utilizes a variety of well-described strategies to evade host innate immune responses, especially affecting host innate immune cells (170), for instance, by interference with TLR signaling in macrophages exerted by targeting sterile alpha and TIR motif containing 1 (SARM1) protein to reduce inflammation and enhance IL-10 production (171). The interaction of Kp with the airway epithelial cells remains the first step of infection that is not well-characterized, and it is likely that Kp utilizes several strategies to compromise host epithelial front-line defenses. Kp secretes ytfL virulence factor that alters cytoskeleton organization in human and mouse airway epithelial cells by disassembling microtubules network (172). Kp translocation through airway epithelium mediated by induction of IFN-λ reduces the host airway epithelial barrier integrity that can facilitate bacterial invasion (173). Furthermore, the encapsulation of Kp with a polysaccharide coat has been considered as a determinant of bacterial virulence. Kp encapsulation limits bacterial binding by the complement component present on the airway mucosal surfaces and reduces bacterial clearance by the host epithelial cells (174). Recent studies showed that encapsulation helps Kp to overcome mouse innate immune defenses in the upper airways primed by microbiota through stimulation of IL-17A expression (175). Interestingly, a recent study indicates that asymptomatic colonization of the gut with MDR-Kp exacerbates pneumonia caused by Pa infection. Altered signaling on the gut–lung axis is caused by MDR-Kp-mediated dysbiosis resulting in reduced production of short-chain fatty acids (SCFAs) and reduced numbers of macrophages and DCs in the lung (176).

*Streptococcus pneumoniae* (Sp) is a respiratory pathogen frequently causing pneumonia (177). The virulence of Sp is associated with secretion of toxin, pneumolysin, exhibiting cytotoxic effect on the host cells accompanied with disintegration of TJs between host epithelial cells for Sp translocation (178). In addition, the cytotoxic effect of pneumolysin is caused by DNA damage associated with the cell cycle arrest (179). Another bacterial virulence factor is pyruvate oxidase SpxB responsible for H_2_O_2_ production and release of pneumolysin (180). Both virulence factors, pneumolysin with contribution of SpxB, were shown to reduce the host innate immune signaling through epigenetic mechanisms. Infection of mice and human airway epithelial cells with Sp led to the activation of the host phosphatase PP1 by its dephosphorylation, which further dephosphorylated histone 3 on serine 10 in the host cells promoting Sp intracellular expansion (181). Sp modifies a component of the cell wall, lipoteichoic acid, to increase its charge to resist host HDPs. Moreover, Sp serine protease PrtA is suggested to cleave HDPs (182, 183). The use of PrtA virulence factor as an antigen has been suggested to develop new vaccines in combination with other antigens to resolve problems of pneumococcal serotype specificity based on the fact that PrtA can also induce protective immunity in certain animal infection models (184). Another study by Biswas et al. showed that group A *Streptococcus*, mainly responsible for necrotizing fasciitis and rarely pneumonia, cleaves the host defense peptide LL-37 with ScpN protease into two shorter peptides that retained bactericidal properties. However, LL-37 cleavage products lost their immunomodulatory properties connected to the activation of P2X7 and EGFR signaling involved in neutrophil bacterial clearance and tissue regeneration, respectively (185, 186).

The respiratory pathogen *Mycobacterium tuberculosis* (Mtb) can bypass host immune defenses by hijacking host cell signaling in macrophages, neutrophiles, and DCs. This way, Mtb creates a favorable intracellular niche where bacteria can replicate and infect other cells or persist for decades in a latent state (187). In the initial step of bacterial colonization, Mtb infects respiratory epithelial cells and macrophages, while 7 days post-infection, the bacteria are present only in macrophages, indicating that bacteria do not replicate in epithelial cells (188). Interestingly, in the zebrafish model, a lipid component of *Mycobacterium marinum* outer membrane phthiocerol dimycocerosate (PDIM) spreads into the epithelial cell membrane and inhibits TLR/MyD88 signaling limiting recruitment of monocytes (189). In humans, Mtb selectively targets uptake by pulmonary macrophages that translocate to the lung interstitium and infect other types of cells. This process is mediated by Mtb virulence factor Esx-1 and the host IL-1β signaling (188). Despite activation of additional host innate immune components connected to the redox signaling, Mtb produces KatG and NuoG virulence proteins that neutralize and inhibit further production of ROS by macrophages and neutrophiles (190, 191). Moreover, CpsA allows Mtb to escape autophagic clearance in macrophages by blocking NADPH oxidase recruitment to the Mtb-containing phagosomes and activation of LC3-associated phagocytosis (192). Mtb creates its own intracellular niche for survival by altering host lipid metabolism, creating the formation of foamy macrophages with characteristic lipid droplets (193). Mechanisms of Mtb evasion and subversion of host immune responses in macrophages and other immune cells are thoroughly described in a recent review (187) that explains why only approximately three Mtb bacterial cells are sufficient for effective colonization of the host (194), indicating a highly advanced Mtb virulence system.

A better understanding of how bacteria breach the host innate defenses on the airway mucosal surfaces to further hijack the host cellular machinery for intracellular survival could result in the development of more efficient therapies. Studies on the molecular mechanism of bacterial virulence factors for host colonization are especially important in the context of immunocompromised individuals suffering from severe infections.

### Viruses

Viruses use a variety of different strategies to compromise the host innate immune defenses and effectively replicate within the host cells or remain in the latent state. Due to the recent pandemic, the majority of research has concentrated on the strategies used by SARS-CoV-2 coronavirus to evade host innate immune responses (195). Yin et al. showed that the host IFN response mediated through intracellular PRRs—MDA5, LGP2, and NOD2—is delayed by several hours in comparison to the kinetics of the viral replication, indicating the viral-strategy-limiting effects of IFNs (196). Moreover, SARS-CoV-2 produces viral endonuclease EndoU that cleaves viral RNA and blocks phosphorylation of STAT1 and STAT2 and their nuclear translocation to inhibit host innate immune responses (197, 198). Subsequently, the virus produces ORF8 protein that disrupts PTMs of host histones promoting closed chromatin state and suppression of host innate anti-viral responses, allowing for viral replication (199). Another virus commonly causing respiratory tract infections, especially harmful for newborns and infants, is RSV, and there is no vaccine available (200). An effective RSV replication within host cells is achieved by initial induction of autophagy and then by blocking autophagosome–lysosome fusion in human airway epithelial cells. This strategy most likely allows to form a replication-favorable niche for the virus inside the autophagosome vesicle (201). The “mimicry” of host chemokines is used to promote infection by RSV, more precisely by the interaction of viral G protein binding receptor with CX3CL1 that facilitates RSV infection (202). The group of Rhinoviruses are the common group of pathogens attacking the host respiratory system causing cold (203). Human rhinoviruses C group (HRV-C) were shown to disrupt epithelial barrier integrity and to alter host metabolism towards glycolysis and fatty acid biosynthesis, facilitating viral replication (204). Rhinoviruses also target another RNA RLRs, such as STING trafficking into viral replication organelles through interaction with PI4P (205). Interestingly, recently, it has been shown that disruption of the circadian clock and expression of immune response genes encoding chemokine receptors (Ccr2, Ccr3, Ccr5, Ccr6, etc.) due to sleep deprivation make mice more susceptible to viral infections, highlighting the importance of environmental factors determining host immune responses against viral infections (206).

### Fungi

Fungal pathogens have developed specific strategies to evade the host immune responses (207). Among them are *C. albicans* triggering oropharyngeal candidiasis, *Aspergillus* ssp. causing pulmonary aspergillosis, and *Pneumocystis* and *Cryptococcus* causing pneumonia (208). *Candida albicans* avoids host responses by neutralization of the host complement components by proteolytic cleavage with aspartyl proteases (209). Moreover, *Aspergillus*, *Mucorales*, and *Candida* spp. cause coronavirus disease (COVID)-associated fungal infections, and aspergillosis was the most prevalent in the group of patients treated with corticosteroids and tocilizumab (210). *Cryptococcus neoformans* evades host innate immune responses, for example, by encapsulation with polysaccharides glucuronoxylomannan (GXM) that inhibits NETs formation (211). Furthermore, *C. neoformans* also produces giant fungal cells, the so-called titan cells, to avoid phagocytosis and killing by ROS, hence considered as a fungal strategy used to establish pulmonary infections (212). Fungal infections are often a complication of the antibiotic treatment because of microbiota dysbiosis, where opportunistic fungal species, such as mentioned *C. albicans*, expand on the host mucosal surfaces forming a biofilm. Those biofilms composed of fungal and bacterial components are hard to eradicate, especially in CF and immunocompromised patients (24); therefore, development of new therapies against such biofilms is needed.

## Modulation of lung innate immunity to overcome pathogen subversion mechanisms

Pathogens quickly adapt to the changing environment, and this includes the presence of antibiotics. The excessive use of antibiotics in the healthcare and animal husbandry has led to the selection for antimicrobial resistance (AMR), which resulted in a global problem that caused 4.95 million of AMR-associated deaths in 2019 (213). Antibiotic resistance occurs in most countries, and many opportunistic pathogens have gained resistance to antibiotics routinely used for the treatment of infections. It is estimated that 63.5% of all infections caused by multidrug-resistant strains were connected to the healthcare (214). Despite of the increasing social awareness on the proper antibiotic use, general reduction in prescribed antibiotics, and a better control of nosocomial infections, the spreading of AMR genes in some pathogens remained unchanged (215, 216). In addition, the development of new antibiotics is a long and costly process, taking several years until the new drug is introduced to the market resulting, in only few developed drugs in the last decades (217). Therefore, alternative strategies for the treatment of infectious diseases have gained more attention. By deciphering molecular pathways of host innate immunity and pathogen’s infection strategies, an opportunity of modulating these mechanisms might result in the development of novel alternative treatment approaches, limiting and/or reducing the use of antibiotics and thereby reducing the selection of AMR strains. Therefore, in our research, we postulate the concept of host-directed therapy (**Figure 2**) executed through the modulation of epithelial and macrophages innate immune responses by enhancing expression of HDPs, improvement of the epithelial barrier integrity, and stimulation of autophagy. Our concept of host-directed therapy can be extended to the restoration of mucociliary clearance and ion balance, training innate immune system *via* controlled TLR stimulation and epigenetic modulation of host innate immune responses. The approach of host-directed therapy does not anticipate replacement of the antibiotics—it is rather a supplementary treatment that can reduce time of antibiotic use in case of persistent infections requiring a long antibiotic treatment, such as tuberculosis and infections requiring prolonged treatment due to poor bioavailability of the drug in the tissue (218). In this section, we will discuss different angles of host-directed therapy for modulation of lung epithelial innate immune responses.

One of the directions in the field is the direct use of HDPs and their analogues, mainly for topical administration (219). Synthetic analogues of HDPs have also been tested in a mouse model of severe lung infection caused by highly virulent *P. aeruginosa*. Nasal instillation of IDR-1002 peptide followed by bacterial infection reduced bacterial count in bronchoalveolar fluid and inflammatory response, indicating therapeutic potential of preventive administration of HDP analogues (220). Although promising, this approach usually uses only one peptide monotherapy, which carries a risk of development bacterial resistance to HDPs, which has been claimed to be limited, however a possible scenario (221). Therefore, the use of HDPs mixture would be a better strategy than a single peptide monotherapy to avoid AMR development, which seems to be highlighted by the fact that in physiological conditions, pathogens usually encounter a mixture of different antimicrobial effectors present on mucosal surfaces. Another aspect is the cost of the production of synthetic peptides and their purification on a bigger scale. Therefore, we suggest an alternative approach by inducing expression of natural HDPs in the epithelial cells and tissue-residing immune cells, which has several advantages in comparison to using synthetic HDPs and their derivatives. First, several antimicrobial effectors are induced, at the same time limiting the risk for the selection of antibiotic resistance. Second, using different innate immune modulators allows for the precise regulation of the HDPs induction and its cessation if needed. Third, the use of different non-peptide inducers for host-directed therapy is expected to reduce the cost of production, making it more accessible for pharmacological use. Those innate immune inducers/modulators were explored by our research group and collaborators and initially included nutrients and bacterial metabolites (like vitamin D3 and butyrate) and further expanded to synthetic chemical compounds (such as aroylated phenylenediamines (APDs)) (**Figure 2**) (218). Among the nutrients, vitamin D3 was shown to induce cathelicidin expression *via* activating vitamin D receptor (VDR) from the group of nuclear receptors binding to the vitamin D responsive elements (VDRE) present in human cathelicidin gene promoter (222, 223). Lung epithelial cells were shown to express the enzyme CYP27B1 involved in conversion of a precursor 25-hydroxyvitamin D3 to an active 1α,25-dihydroxyvitamin D3 that induced cathelicidin expression, indicating the importance of the vitamin D3 levels for mucosal antimicrobial defenses (30). Interestingly, VDREs are absent in mouse *CRAMP* promoter, making the translation of research on vitamin D3 inducer to mouse models difficult. For that reason, the novel transgenic model of humanized mice was established recently and contains human VDRE in the promoter of mouse *CRAMP* gene, opening a new avenue for the studies on vitamin D3 (224). Another inducer of cathelicidin expression in lung epithelium and macrophages is butyrate, a short-chain fatty acid produced by microbiota commensal bacteria from Firmicutes phylum (225). Butyrate constitutes the primary energy source for colonocytes, exerting local immunomodulatory effects on the colonic epithelia, tissue residing immune cells, and gastrointestinal (GI)-distant organs because butyrate is distributed with blood similar to other microbiota metabolites (218, 226). Butyrate is known from its strong odor; therefore, 4-phenylbuturate (hereafter phenylbutyrate or 4-PBA) was used in the studies as an odorless butyrate derivative, which is an FDA-approved drug for treatment of the urea cycle disorders used as ammonia scavenger (227). Phenylbutyrate was shown to induce cathelicidin expression in the airway epithelium (**Figure 2**) and induced autophagy in Mtb-infected macrophages through induction of LL-37 acting *via* P2X7 receptor (228, 229). Phenylbutyrate also counteracted the downregulation of cathelicidin expression in lungs of *Shigella*-infected rabbits, mitigating pathogens strategy of effective host colonization (230, 231). Phenylbutyrate is an inhibitor of histone deacetylases (HDACs)—histone-modifying enzymes facilitating chromatin opening state by acetylation of histones. Although *CAMP* gene expression was induced by phenylbutyrate, the increased acetylation of histone 3 and histone 4 was not observed in the promoter of *CAMP* gene (228). Therefore, the detailed mechanism of *CAMP* induction by phenylbutyrate remains unknown, perhaps requiring analysis of more specific histone modifications in *CAMP* promoter. The combination of 1,25-dihydroxyvitamin D3 and 4-PBA is so far the most potent inducer of cathelicidin expression in the human model of the lung epithelium, most likely by combing transcriptional regulation of vitamin D3 and epigenetic modulation by 4-PBA. Importantly, the combination of vitamin D3 and 4-PBA has clinical translational potential and was shown to be effective in the clinical trial of the Mtb treatment combined with standard antibiotics. Patients with tuberculosis receiving phenylbutyrate and vitamin D3 showed faster clearance of Mtb in sputum samples than the group receiving placebo, indicating beneficial effects of host-directed therapy combined with antibiotics (232, 233).

Moreover, in respect to the lung epithelium, other inducers of HDPs were tested from a novel class of compounds called aroylated phenylenediamines (APDs) (**Figure 2**). Two synthetic APD-compounds, derivatives of Entinostat, which is another HDAC inhibitor known to induce *CAMP* gene expression, were shown to induce expression of antimicrobial effectors cathelicidin, calprotectin, lipocalin, and defensins. Of note, APDs were less toxic than Entinostat and did not induce proinflammatory responses (32, 234). Furthermore, innate immune inducers 4-PBA, vitamin D3, and APD compound were able to sensitize MDR *K. pneumoniae* to conventional antibiotics. The intracellular bacterial killing mechanism in infected macrophages was cathelicidin dependent for 4-PBA and vitamin D3 and ROS dependent for APD compound (235). This approach presents another angle on host-directed therapy, utilizing modulation of host innate immune responses and sensitization of multidrug-resistant pathogens to conventional antibiotics. Moreover, APD-compound induced autophagy in the differentiated lung epithelial cells through the epigenetic modulation of the H3K27 acetylation and AMP-activated protein kinase (AMPK) signaling (236). The preventive treatment of differentiated lung epithelial layers with APD compound enhanced the epithelial barrier integrity provided by tight junction’s proteins occludin and ZO-1 when differentiated monolayer cells were challenged with *P. aeruginosa*-conditioned medium (32). Interestingly, one of the most often prescribed antibiotics, azithromycin, was shown independently of its microbicidal properties to modulate host’s epithelial cell responses by strengthening epithelial integrity in the lung (**Figure 2**). This can be paradoxically considered as a positive side effect of azithromycin treatment that benefits cystic fibrosis patients by improving their condition (237). Azithromycin was shown to enhance epithelial barrier integrity by increasing trans-epithelial electrical resistance (TEER) and counteract the disruptive effect of *P. aeruginosa*-conditioned medium (238). Furthermore, azithromycin was shown to have a barrier protective effect and counteract proinflammatory response inflicted on the bronchial epithelia by cyclical mechanical stress during mechanical ventilation generated by a cyclical pressure air–liquid interface device (CPAD) (239). The supporting evidence suggest that the protective effect of azithromycin on lung epithelia is exerted by increased lamellar body formation and stimulation of epidermal differentiation (240). However, azithromycin is an antibiotic; therefore, prolonged use of azithromycin to strengthen the epithelial barrier is restricted because of the risk for antibiotic resistance development. Importantly, the mechanism behind strengthening of the lung epithelial barrier integrity by azithromycin and APDs remain unknown, and it is a subject of the ongoing research (32, 235, 236, 239, 240). On the contrary, vitamin D3 did not have this functional effect on the epithelial barrier strengthening while administered over the course of lung epithelial differentiation. Instead, it led to thickening of the lung epithelial layer *in vitro* displaying features of squamous metaplasia, indicating that this effect in the lung is tissue specific and is contrary to what has been observed in the gut (241). The group of Jun Sun showed that vitamin D3 regulates expression of a tight junction protein claudin-2, demonstrating the potential of VDR to regulate gut epithelial barrier integrity (242, 243). Different effects of innate immune inducers have been described depending on the tissue type, which may suggest possible epigenetic regulation of the epithelial barrier function. This concept seems to be additionally supported by the fact that many different respiratory tract diseases are associated with changes in the expression of histone-modifying enzymes, such as increased expression of histone deacetylases, HDACs (1 and 9) and sirtuins (SIRT6 and 7), in asthmatic bronchial epithelial cells. Inhibition of the HDACs with quisinostat (JNJ-26481585) in bronchial epithelial cells from asthmatic patients and allergic rhinitis improved epithelial barrier integrity by affecting expression and reorganization of TJs (149, 244). Recently, azithromycin was shown to attenuate wheezing in patients recovering from pulmonary inflammation. Azithromycin treatment helped to reduce time of wheezing for those patients, and these changes were associated with reduced expression of EZH2 (histone methyltransferase responsible for methylation of H3K27me3), reduced methylation of H3K27me3, and reduced expression of p65, suggesting that azithromycin exerts anti-inflammatory properties through epigenetic regulation (245). Overall, epigenetic modulation is part of the natural physiological regulation of innate immune responses in epithelial tissues and immune cells exerted by microbiota-produced metabolites of dietary products, highlighting the future directions for modulation of host innate immune responses through epigenetic therapies utilizing natural and synthetic epigenetic modulators. It is important to keep in mind that epigenetic therapies may exert off-target effects on other tissues, making the precise regulation difficult to control. However, transient epigenetic modulation over a short period of time with relatively rapidly degraded synthetic compounds and natural products, such as microbiota-produced butyrate, seems to be a reasonable approach.

In line with host-directed therapy is the concept of trained immunity responses for the treatment and prevention of respiratory tract infections. A type of immunotherapy with Broncho-Vaxom, a lyophilizate of the dead bacterial strains causing respiratory tract infections, such as *K. pneumoniae*, *S. aureus*, *Streptococcus pyogenes*, and *Neisseria catarrhalis*, is used to train immune responses in patients suffering from recurrent infections of upper and lower respiratory tract (**Figure 2**). Broncho-Vaxom showed efficiency in reducing recovery time and course of disease in pediatric patients with recurrent respiratory tract infections (246, 247). Beneficial effects of Broncho-Vaxom^®^ (OM-85 BV) on human sinonasal epithelial cells were mediated through stimulation of the taste-receptor T2R signaling, leading to the production of nitric oxide (NO) responsible for direct bacterial killing and increased ciliary beating (248). Treatment of bronchial epithelial cells with bacterial lysates of Broncho-Vaxom protects epithelia from SARS-CoV-2 entry by reducing expression of host receptors used by the virus such as angiotensin-converting enzyme 2 (ACE2) (249, 250), suggesting that this form of host-directed therapy can be used as a preventive strategy to limit acute respiratory disease, for example in constantly exposed health workers (ClinicalTrials.gov Identifier: NCT04496245).

The concept of trained immunity refers to innate immunological memory previously attributed only to the adaptive immune responses. Trained immunity boosts secondary responses to infections or sterile inflammation after initial contact with the stimuli for the next faster and more efficient host responses. Although trained immunity responses are T- and B-cell independent, they complement the adaptive immune responses to maximize chances for the host survival. The innate immunological memory can be achieved by stimulation of innate immune cells, such as macrophages, NK cells, DCs, fibroblasts, and tissue-specific stem cells (251, 252). The primary example of trained immunity is *Bacillus* Calmette–Guérin (BCG) vaccination used routinely in vaccination against tuberculosis and shown to reprogram hematopoietic stem cells to differentiate towards monocytes/macrophages with enhanced capabilities of Mtb clearance. Unlike subcutaneous BCG vaccination, intravenous BCG administration enhanced myelopoiesis and rewired epigenetic program in bone-marrow-derived macrophages connected to the changes in the H3K27ac and H3K4me3 marks for more efficient Mtb clearance that was IFNγ dependent (253). Microbiota metabolites have also been identified as elicitors of trained immunity. One of them is butyrate that affects the trajectory of antimicrobial responses in macrophages by imprinting antimicrobial program in differentiating macrophages through HDAC3 inhibition, resulting in the induction of calprotectin and enhanced bacterial clearance through autophagy (254). Another microbiota metabolite, deoxycholic acid (DCA), a secondary bile acid detected in the bloodstream, is shown to enhance differentiation of granulocyte-monocyte precursors in the bone marrow through epigenetic alterations, providing innate protection against parasite *E. histolytica*. Interestingly, DCA in sera of children from Bangladesh who previously have documented history of amebiasis had lower levels of DCA than those who never suffered from infection (255). Trained immunity was also described in the lung in the context of the allergic inflammatory memory inflicted on basal lung stem cells by IL-4 and IL-13 exposure. The chronic exposure of respiratory epithelial progenitor cells to the inflammatory type 2 immune responses shifts their differentiation program causing epithelial barrier dysfunction observed in chronic allergic diseases, resulting in rhinosinusitis and taking more extreme form of nasal polyps (256). Overall, trained immunity can be achieved by exposition of tissue-specific stem cells and their progenitors to different stimuli imprinting innate immune responses through metabolic and epigenetic reprogramming (257).

## Future perspectives

Innate immunity of the lung epithelial surfaces is a complex system working together with the adaptive immune responses for host defense and survival. Many aspects of the innate immune regulation and the link between innate and adaptive immune responses remain to be further elucidated. These include characterization of the role of new types of cells in the respiratory epithelium identified by single-cell RNA sequencing in shaping local innate immune responses and epigenetic modulation of such responses. An exciting avenue is the exploration of how different microbiota metabolites shape host immune responses in lung epithelial cells for better protection against pathogens with defining molecular mechanism that can be further extended to the development of the synthetic compounds for host-directed therapy. Defining pathogen’s effectors subverting host innate immune responses, especially in the context of compromised host innate immunity, include future steps of interest for the development of new treatment strategies. Furthermore, the concept of trained immunity mainly characterized in respect to immune cells remains to be further investigated in lung epithelial cells answering the questions on programming our epithelial cells, how innate immune memory confers to better host protection mechanisms, and whether there are any links to the development of chronic inflammatory diseases with defining environmental stimuli that shape such responses.

## Author contributions

The conceptualization of the manuscript was based on ITM’s PhD dissertation thesis from year 2021. ITM wrote and edited the manuscript including figures created with BioRender.com under license agreement. GHG commented and edited the manuscript. All authors contributed to the article and approved the submitted version.
